# Analysis of tomato gene promoters activated in syncytia induced in tomato and potato hairy roots by *Globodera rostochiensis*

**DOI:** 10.1007/s11248-012-9665-4

**Published:** 2012-11-06

**Authors:** A. Wiśniewska, J. Dąbrowska-Bronk, K. Szafrański, S. Fudali, M. Święcicka, M. Czarny, A. Wilkowska, K. Morgiewicz, J. Matusiak, M. Sobczak, M. Filipecki

**Affiliations:** 1 Department of Plant Physiology, Faculty of Agriculture and Biology, Warsaw University of Life Sciences (SGGW), Nowoursynowska 159, 02-776 Warsaw, Poland; 2 Department of Plant Genetics, Breeding and Biotechnology, Faculty of Horticulture and Landscape Architecture, Warsaw University of Life Sciences (SGGW), Nowoursynowska 159, 02-776 Warsaw, Poland; 3 Department of Botany, Faculty of Agriculture and Biology, Warsaw University of Life Sciences (SGGW), Nowoursynowska 159, 02-776 Warsaw, Poland; 4 Present Address: Systématique, Adaptation, Evolution (SAE), Université Pierre et Marie Curie, UMR 7138 (UPMC CNRS IRD MNHN), bâtiment A, 7, quai St. Bernard, 75005 Paris, France

**Keywords:** Gene promoter, *Globodera rostochiensis*, Hairy roots, Nematode, Tomato, Syncytium

## Abstract

The potato cyst nematode (*Globodera rostochiensis*) induces feeding sites (syncytia) in tomato and potato roots. In a previous study, 135 tomato genes up-regulated during *G. rostochiensis* migration and syncytium development were identified. Five genes (*CYP97A29*, *DFR*, *FLS*, *NIK* and *PMEI*) were chosen for further study to examine their roles in plant–nematode interactions. The promoters of these genes were isolated and potential *cis* regulatory elements in their sequences were characterized using bioinformatics tools. Promoter fusions with the β-glucuronidase gene were constructed and introduced into tomato and potato genomes via transformation with *Agrobacterium rhizogenes* to produce hairy roots. The analysed promoters displayed different activity patterns in nematode-infected and uninfected transgenic hairy roots.

**Electronic supplementary material:**

The online version of this article (doi:10.1007/s11248-012-9665-4) contains supplementary material, which is available to authorized users.

## Introduction

Sedentary root endoparasitic (root-knot and cyst forming) nematodes can have a serious negative impact on crop production. Both types of nematode induce specialized nematode feeding sites (NFS) and their life cycles and parasitic habits are well characterized (Williamson and Hussey 1996; Sobczak and Golinowski 2011). The parasitic second-stage juvenile migrates through the cortex towards the vascular cylinder. It then selects an initial syncytial cell among inner cortical cells (*Globodera* sp.) or cambial cells (*Meloidogyne* sp. and *Heterodera* sp.) from which to form the NFS. The properly formed feeding site provides each juvenile with permanent nutrient delivery and allows them to progress to maturity, i.e. sedentary egg-laying females or motile males.

Traditional methods of plant protection against nematodes (fallow periods, inundation, crop rotation, and nematode repelling soil treatments) are costly and not sufficiently effective. On the other hand, anti-nematode chemicals can cause environmental damage. Biological control methods (e.g. nematopathogenic fungi) are still under development (Yan et al. 2011). Similarly, biotechnological methods focused on transgenic plants and classical breeding methods based on natural host resistance genes have yet to fulfil their potential. The resistance mediated by tomato *H1* (Bakker et al. 2004) is effective only in the case of the Ro1 pathotype of *Globodera rostochiensis* and the resistance against *Globodera pallida* provided by the *Gpa2* gene (van der Vossen et al. 2000) has been overcome by this pathogen (Gommers and Bakker 1993). The tomato *Hero* gene provides different levels of resistance to all pathotypes of *G. rostochiensis* and *G. pallida* (Ernst et al. 2002). The introduction of *Hero* into a susceptible tomato cultivar caused an appreciable decrease of the number of developing nematode females, but no resistant reaction was observed in transgenic potato plants carrying the *HeroA* gene (Sobczak et al. 2005). The potato *Gro1*–*4* and *Hs1* ^*pro*−1^ genes, which provide resistance against the Ro1 pathotype of *G. rostochiensis* (Paal et al. 2004) and *Heterodera schachtii* (Cai et al. 1997), respectively, were not effective when transferred alone into different plant species. When transferred to tomato, the *Mi* gene originating from *Solanum peruvianum* conferred resistance to root-knot nematodes *Meloidogyne incognita*, *Meloidogyne javanica* and *Meloidogyne arenaria*, but not to *Meloidogyne hapla* (Ammati et al. 1985; Hadisoeganda and Sasser 1982, Kaloshian et al. 1996). Resistance controlled by this gene was found to be unstable at higher temperatures (Ammati et al. 1986; Dropkin 1969). Milligan et al. (1998) confirmed that one of two candidate genes in the *Mi* locus (*Mi*-*1.2*) is responsible for tomato resistance to three *Meloidogyne* species.

One of the most promising biotechnological approaches to the production of nematode-resistant plants is the specific localization of anti-nematode products within a NFS. Several strategies may be used to enhance plant resistance to these pathogens, e.g. dsRNA targeted against plant or nematode genes, or the expression of anti-nematode products. The application of this type of approach using constitutive promoters can produce unintended effects in uninfected plant organs. Therefore, precise spatial and temporal control of transgene expression is very important and this can be achieved by carefully selecting the promoter regions used in gene constructs. When attempting to combat plant nematode parasitism, transgene expression should be restricted to the inedible roots (e.g. tomato, potato), or even strictly to the NFS, particularly in plant species where the roots are economically important (e.g. beet). To date, there have been no reports of native promoters that are active only in NFS (after nematode attack) and many candidate genes need to be analysed in order to obtain the ‘ideal’ promoter for this purpose. It may be necessary to isolate and combine *cis* regulatory elements of various origin to produce an artificial promoter that is strongly activated in NFS.

As no single method can produce solid plant immunity to nematode attack, a combined approach is required, e.g. resistance genes in combination with mechanisms of disturbing the plant–nematode interaction. However, the latter strategy should not disrupt the metabolism of non-infected plant cells. Nematodes have evolved sophisticated strategies for exploiting their host plants based on natural and non-specific plant mechanisms. The modulation of plant cell metabolism by parasitic nematodes is achieved by secretions from nematode glands, which evoke changes in host gene expression and lead to the establishment and maintenance of the NFS (Abad and Williamson 2010). Secreted proteins with different functions, called effectors, can subtly but precisely manipulate plant cell metabolism (Haegeman et al. 2012). Blocking of nematode-induced plant molecular processes promoting nematode development can be achieved by transgene expression targeting these specific mechanisms.

Transformed hairy roots produced by infection of plant tissues with the gram-negative bacterium *Agrobacterium rhizogenes* have been used in studies on plant–nematode interactions, including plant promoter analysis (Hansen et al. 1996; Wang et al. 2007) and gene function analysis (Gal et al. 2006; Huang et al. 2006; Li et al. 2010a; Plovie et al. 2003; Urwin et al. 1995).

We have previously identified 226 tomato genes that show modified expression during *G. rostochiensis* migration and syncytium development (Swiecicka et al. 2009; unpublished data). For the present study, we selected the genes *CYP97A29*, *DFR*, *FLS*, *NIK* and *PMEI* that encode putative proteins with homology to other known proteins related to defense reactions, and that show up-regulation during the first days post infection by *G. rostochiensis* (Swiecicka, unpublished data). The product of the *CYP97A29* gene belongs to a family of P450 monooxygenases, which are involved in the biosynthesis of many different compounds, i.e. flavonoids, phenolic esters, coumarins, glucosinolates, as well as antioxidants and defence compounds (Kahn and Durst 2000). Moreover, *CYP97A29* encodes carotenoid β-hydroxylase, which participates in lutein biosynthesis in tomato leaves and fruit (Stigliani et al. 2011). Cytochrome P450 genes from other plant species are known to be involved in defence responses against microbial pathogens, e.g. pepper *CaCYP450A* (Hwang and Hwang 2010), *Arabidopsis* *CYP82C2* (Liu et al. 2010) and wheat *CYP709C1* (Li et al. 2010b). The *DFR*, *FLS*, *NIK* and *PMEI* genes encode a putative dihydroflavonol-4-reductase (DFR), flavonol synthase/flavanone 3-hydroxylase (FLS), protein kinase domain-containing protein (NIK—nematode induced kinase protein) and a plant invertase/pectin methylesterase inhibitor domain-containing protein (PMEI), respectively, which have not previously been described in tomato. DFR and FLS are enzymes participating in flavonoid biosynthesis. Flavonoids are plant secondary metabolites involved in plant defences against pathogenic microorganisms, but they also participate in symbiotic plant–microbe interactions (Wasson et al. 2006). These compounds are known to accumulate in plant tissues in response to nematodes (Hutangura et al. 1999; Jones et al. 2007). PMEs (pectin methylesterases) are produced by pathogenic microorganisms during plant infection and in symbiotic plant–microbe interactions (Lievens et al. 2002). Hewezi et al. (2008) showed that PME3 from *Arabidopsis thaliana* is a target for the cellulose binding protein (HgCBP) of *H. schachtii*, and this interaction probably facilitates cyst nematode parasitism. PME activity may be regulated by either differential expression or posttranslational control by PME protein inhibitors (PMEIs) (Giovane et al. 2004). The overexpression of two inhibitors, AtPMEI-1 and -2, resulted in a decrease in PME activity and an increase in resistance to fungus *Botrytis cinerea* in *Arabidopsis* (Lionetti et al. 2007). Recombinant and purified pepper CaPMEI1 protein exhibited in vitro antifungal activity against three plant pathogenic fungi (*Fusarium oxysporum* f.sp*. matthiole, Alternaria brassicicola* and *B. cinerea*), while *CaPMEI1*-silenced pepper plants showed enhanced susceptibility to *Xanthomonas campestris* pv. *vesicatoria* infection (An et al. 2008). Transgenic *Arabidopsis* plants overexpressing CaPMEI1 displayed enhanced resistance to *Pseudomonas* *syringae* pv. *tomato* DC3000, but not to *Hyaloperonospora parasitica* (An et al. 2008). To date there is no clear evidence for the direct involvement of the genes selected for this study (or their orthologues) in plant–nematode interactions.

The aim of this study was to isolate the promoter regions of the aforementioned genes and to analyse their activity in tomato and potato roots, before and during nematode parasitism.

## Materials and methods

### Promoter isolation and cloning

Genes were selected from a set of tomato genes that were shown to be up-regulated after *G. rostochiensis* infection by Swiecicka et al. (2009) (Table S1). The 5′ upstream regions of these genes were amplified using a BD Advantage™ Genomic PCR kit (BD Biosciences Clontech) from adaptor-ligated tomato genomic libraries prepared by the GenomeWalker™ protocol (BD Bioscences Clontech). Genomic DNA was isolated from frozen tomato leaves using the CTAB method. The quantity and quality of the gDNA were assessed spectrophotometrically and by gel electrophoresis. The gDNA was digested in separate reactions with a panel of four restriction endonucleases cleaving 6-bp recognition sequences to leave a blunt end (*Dra*I, *Eco*RV, *Pvu*II, *Stu*I). A GenomeWalker adaptor DNA was ligated to the ends of fragments in each digest mixture to produce four adaptor-ligated libraries. Genomic sequences were amplified from these libraries by nested PCR using primers designed to the 5′ ends of the respective cDNAs (Table S1) in combination with adaptor primers (AP1 and AP2). The gene-specific primers were designed using the OLIGO program (Primer Analysis Software ver. 6.54, Molecular Biology Insight Inc., USA). The PCR products were cloned in vector pCRII-TOPO (Invitrogen) and sequenced. To identify potential *cis*-acting regulatory elements, the promoter fragment sequences were analysed with the PLACE program (Higo et al. 1999; http://www.dna.affrc.go.jp/PLACE/signalscan.html). The obtained promoter sequences also contained the 5′ UTR (untranslated region) of the genes.

### Reporter gene construct preparation

To prepare constructs in which the isolated promoters were fused with the β-glucuronidase (*gusA*) reporter gene, the fragments were subcloned into a modified pCAMBIA1381Z binary vector (http://www.cambia.org) containing the kanamycin resistance gene (*nptII*) instead of the hygromycin resistance gene (*hpt*). To facilitate subcloning, promoter fragments were amplified using primers containing added restriction sites (Table S2), digested with these restriction endonucleases and ligated to the vector that had been cleaved with the same enzymes. The desired constructs were transferred into *A. rhizogenes* ATCC 15834 by electroporation (MicroPulser, Bio-Rad).

### Plant transformation and infection with *G. rostochiensis*

Tomato (*Lycopersicon esculentum* Mill. cv. Money Maker) and potato (*Solanum tuberosum* L. cv. Desiree) plants were used in the study. Tomato and potato hairy roots were obtained as described by Hwang et al. (2000). Cotyledons or hypocotyls were excised from 8- to 10-day-old tomato seedlings and the tips of the former were removed before immersion in *A. rhizogenes* suspension for 30 min. The cotyledon explants were blotted on sterilized filter paper to remove excess bacteria and then transferred onto solid 1/2 MS medium (Murashige and Skoog 1962) containing 2 % sucrose and 0.8 % agar. Three days later, the cotyledons were transferred onto fresh MS medium containing 75 mg L^−1^ kanamycin and 200 mg L^−1^ timentin, and left for 7–10 days at 26 °C in darkness. During this incubation period, hairy roots become visible on the surface of the explants. In the case of potato, stem segments and leaves were used for transformation. As negative controls, explants of both species were also transformed by the wild-type strain of *A. rhizogenes* or *A. rhizogenes* carrying the unmodified pCAMBIA1381Z vector (*gusA* gene lacking a promoter sequence). Hairy roots grew on selective medium in darkness and were tranfered on the fresh medium every 3 weeks as 2–3 cm root explants.

After 14 days incubation on selective medium, subclonned hairy roots from independent transformation events (ITE) were transferred to fresh antibiotic-free MS medium supplemented with 2 % sucrose and 1.5 % agar (pH 6.2) (three root explants per Petri dish). After a further 14–21 days, the roots were inoculated with about 200 freshly hatched sterile second-stage juveniles of *G. rostochiensis* Woll. (pathotype Ro1) per Petri dish. The juveniles were obtained from dry cysts as described by Goverse et al. (2000).

### GUS activity assay

Histochemical detection of GUS activity was performed according to the method of Jefferson et al. (1987). The root samples were incubated in 1 mM X-Gluc in 50 mM NaH_2_PO_4_ pH 7.2 at 37 °C for 16 h in darkness. Hairy roots obtained after inoculations with wild-type *A. rhizogenes* without a binary vector or with *A. rhizogenes* carrying unmodified pCAMBIA1381Z were used as controls. GUS activity was examined in newly emerged hairy roots 7 days after subculturing. GUS activity was examined at 7, 14, 21 and 90 dpi (days post infection). The numbers of analysed hairy roots derived from ITEs for each time point are shown in Table S3. At least three independent ITS were used for the experiment where each ITS was represented by number of clones on separate plates and one of these clones (usually three roots containing lateral roots per plate) was used for each time point. The analysis of uninfected and infected ITEs was repeated 3–5 times.

### RNA isolation and RT-PCR

Total RNA was isolated according to method of Chomczynski and Sacchi (2006) from 100 mg of 0.5 mm-long root-tip segments containing apical meristems, roots without meristems collected from a minimum of five 14-day-old tomato plants and root segments containing syncytia at 14 dpi. Prior to RT-PCR, the RNA preparations (15 μg) were treated with RNase-free DNase I (Fermentas) to remove any genomic DNA contamination. First-strand cDNA was synthesized from 0.2 μg of DNase-treated RNA using a RevertAid™ First Strand cDNA Synthesis Kit (Fermentas). For RT-PCR, 1 μl of the cDNA preparations was used in each 20 μl reaction with gene-specific primers. The primer sequences and annealing temperatures are listed in Table S4. The optimal number of PCR cycles was determined for each of the primer pairs and all amplifications were carried out using 29 cycles. A fragment of the constitutively expressed tomato *UBI3* gene (Hoffman et al. 1991) was amplified in control PCRs. As template for a DNA control, 0.2 μg of DNase-treated RNA were used.

## Results

### Cloning and characterization of promoter regions

In order to characterize the regulation of the five selected tomato genes more precisely, the upstream regions of the *CYP97A29*, *DFR*, *FLS*, *NIK*, and *PMEI* genes were amplified by nested PCR, cloned and sequenced. The length of the obtained promoter fragments and 5′ UTR regions, and the putative *TATA*-box positions are shown in Table 1. The sequences upstream of the start codon were screened for *cis* regulatory elements using the PLACE algorithm. Sequence motifs related to pathogen, growth regulator and abiotic stress responses constituted about 22 % (19.5–29 %) of all identified *cis* elements. The identified putative pathogen response *cis* regulatory elements are presented in Table S5.

**Table 1:** Characteristics of the isolated promoter regions

Gene	Acc. no.^a^	Length of promoter sequence (bp)^b^	Length of 5′ UTR (bp)	Putative positions of TATA-box^c^
*CYP97A29*	HE795780	1,764	131	−37
*DFR*	HE795781	988	82	−171
*FLS*	HE795779	1,652	28	−94, −110
*NIK*	HE795778	1,058	187	−80
*PMEI*	HE795782	1,329	114	−28, −30

^a^EMBL nucleotide sequence database

^b^From the 5′ end of the obtained promoters to the ATG start codons

^c^Positions relative to 5′ end of 5′ UTR

### Establishment of hairy root cultures

To examine the specificity of the analysed promoters during nematode infection, the hairy root system was used. Cotyledon explants (Fig. S1a) appeared to be more suitable than hypocotyl explants for the production of tomato hairy roots, giving greater numbers of transformed roots and a higher root growth rate. In the case of potato (Fig. S1b), stem explants were more efficient than leaf explants for the production of hairy roots.

### Activation of promoter regions in uninfected hairy roots

Depending on the analysed promoter, GUS activity was examined in hairy roots obtained from between 3 and 9 ITE (Table 2). Hairy roots obtained by transformation with wild-type *A. rhizogenes* or *A. rhizogenes* carrying unmodified vector pCAMBIA1381Z(k) served as controls.

**Table 2:** GUS activity produced by the analysed promoter regions in tomato and potato hairy roots

Gene name	Location of GUS activity in tomato	Number of analysed ITEs	Location of GUS activity in potato	Number of analysed ITEs
*CYP97A29*	In some young root primordia, root elongation and/or differentiation zones and/or stele of CRP	4	Root elongation zone or CRP or whole roots	3
*DFR*	CRP, root elongation zone, or root elongation and differentiation zones	7	In most cases, whole roots, but sometimes without meristems, or only in root elongation zone and/or its meristems^a^	7
*FLS*	Stele of CRP or root elongation zone	5	Whole roots or CRP^a^	4
*NIK*	Stele, root-tip meristems and root primordia	3	Whole roots^a^	3
*PMEI*	Root meristems, primordia, or elongation zone, or stele of CRP, root-hairs	7	Whole roots or root tips (meristems, elongation and differentiation zones), primordia	9

*CRP* central root part, i.e. root fragment without the root base and meristem

^a^GUS activity was lower than in tomato roots

**Fig. 1:**
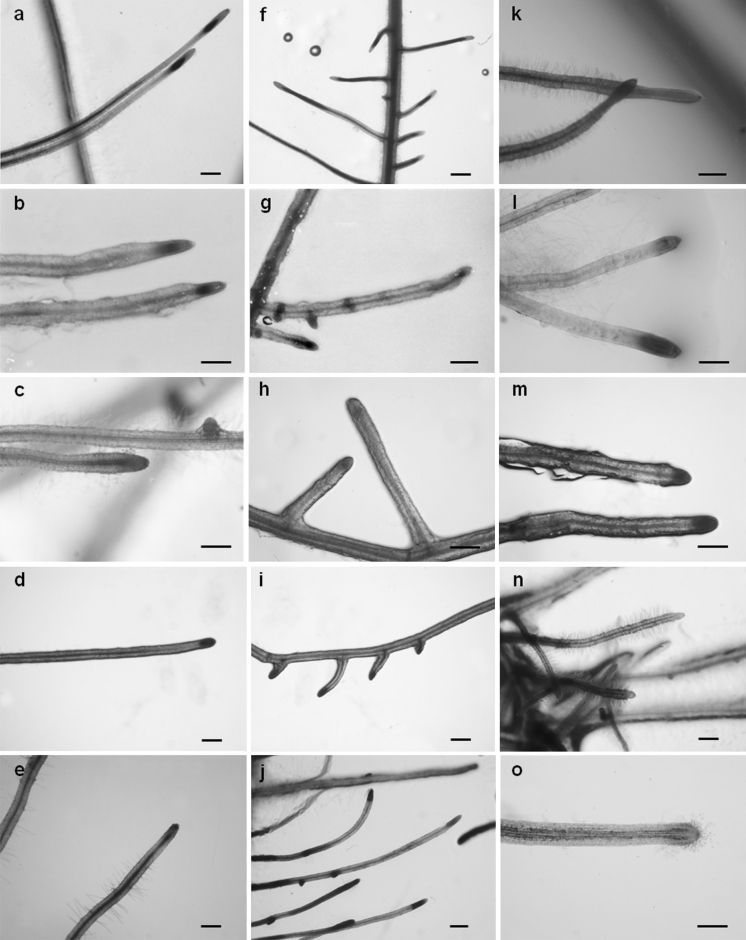
Activity of promoters in uninoculated tomato (**a**–**j**) and potato (**k**–**o**) hairy roots. GUS staining of transgenic lines carrying the promoters of the genes *CYP97A29* (**a**, **f** and **k**), *DFR* (**b**, **g** and **l**), *FLS* (**c**, **h** and **m**), *NIK* (**d**, **i** and **n**) and *PMEI* (**e**, **j** and **o**). Details in Table 2. *Scale bars* 0.5 mm

No GUS activity was observed in any control hairy root line (data not shown). GUS activity was detected in uninfected tomato and potato hairy roots obtained using all of the promoter fusions (Fig. 1). However, none of the analysed lines showed any wound-related GUS activity. The patterns of activity appeared to be similar for all of the analysed promoters. GUS staining was observed in meristems, lateral root primordia, the epidermis and stele (along the whole root or only in sectors). In the oldest parts of hairy roots, no GUS activity was usually observed where lateral roots emerged. GUS activity was detected in secondary and tertiary lateral roots. Details are presented in Table 2. Most, but not all of the hairy roots cultured under antibiotic selection showed GUS activity.

### Changes in promoter activity in hairy roots following infection with *G. rostochiensis*

GUS activity was examined in hairy roots 7, 14, 21 and 90 days after inoculation with juveniles of *G. rostochiensis*. After infection, the growth of syncytia as well as the development of nematodes was observed (Fig. S2). No GUS activity was found in control hairy roots obtained by transformation with wild-type *A. rhizogenes* or a strain carrying unmodified pCAMBIA1381Z(k), following *G. rostochiensis* infection.

**Fig. 2:**
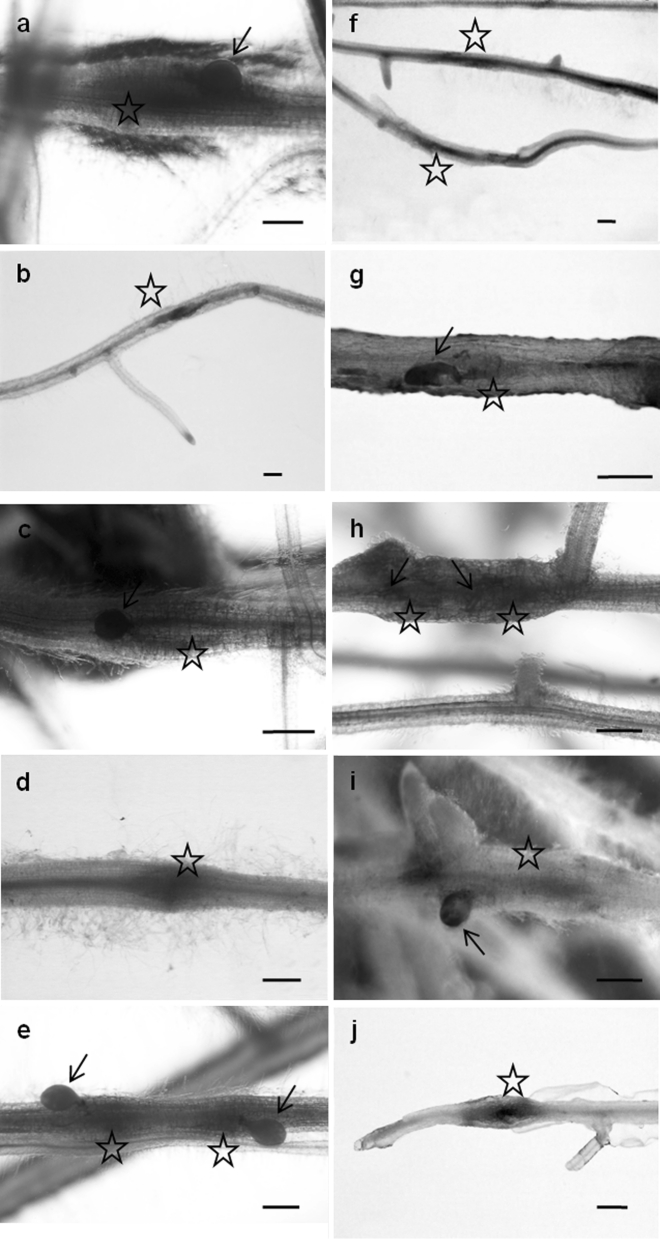
Activity of *CYP97A29*, *DFR*, *FLS*, *NIK* and *PMEI* promoters in *G. rostochiensis* NFS induced in tomato (**a**–**e**) and potato (**f**–**j**) hairy roots. *CYP97A29* at 21 (**a**) and 7 (**f**) dpi. *DFR* at 14 (**b**) and 21 (**g**) dpi. *FLS* at 21 dpi (**c** and **h**). *NIK* at 14 (**d**) and 90 (**i**) dpi. *PMEI* at 21 dpi (**e** and **j**). Numbers of analysed hairy roots are shown in Table S3. Syncytium (*star*), nematode (*arrow*). *Scale bars* 0.5 mm

Necrosis of root tissues was observed during the migration of juveniles, but no GUS activity was detected in cells located next to these necrotic areas at 7 dpi. Where a juvenile induced a NFS, GUS activity was observed at 7 dpi in syncytia, but only in hairy roots containing the *NIK* or *CYP97A29* promoter constructs, in tomato and potato, respectively (Fig. 2f). However, the regulatory regions of all the analysed genes produced GUS activity in older syncytia (at 14–21 dpi) in both plant species (Fig. 2). Moreover, the promoter activities were not changed in other parts of the roots after nematode infection. At 90 dpi, GUS activity in syncytia was detected only in potato hairy roots carrying the *NIK* promoter construct (Fig. 2i).

**Fig. 3:**
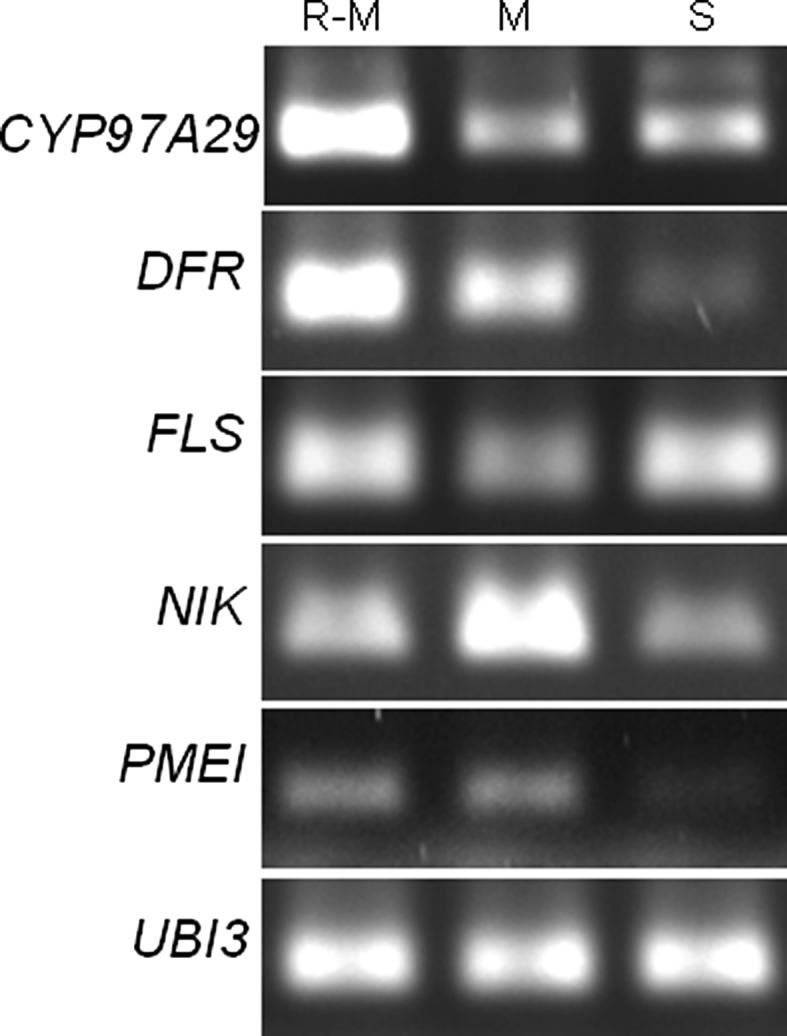
Semi-quantitative RT-PCR analysis of *CYP97A29*, *DFR*, *FLS*, *NIK* and *PMEI* transcript levels in *G. rostochiensis*-infected and uninfected tomato roots. The tomato *UBI3* gene was used as a control. *M* root-tip meristems of uninfected roots. *R*–*M* uninfected roots without root-tip meristems. *S* root segments with syncytia at 14 dpi

It was noted that GUS activity was sometimes absent from syncytia in different hairy root lines infected with *G. rostochiensis*. To examine this phenomenon, the GUS staining of syncytia in tomato hairy roots was evaluated at 21 dpi (Table S6). On average, about 40 % of the developed syncytia showed no blue staining and there was no relationship between the lack of GUS activity and the promoter construct used or the sex of the attached nematode.

### Expression profiles of *CYP97A29*, *DFR*, *FLS*, *NIK* and *PMEI* genes

Using the cDNA-AFLP method, Swiecicka et al. (2009) found that five genes analysed in the present study were up-regulated in *G. rostochiensis*-infected roots from 1 to 14 dpi, but the highest transcript accumulation was observed at 1 and/or 3 dpi. To verify this finding and to confirm our results obtained using promoter-*gusA* fusions, the expression of the *CYP97A29*, *DFR*, *FLS*, *NIK* and *PMEI* genes in infected and uninfected tomato plants was studied by semi-quantitative RT-PCR. RNA was isolated from tomato seedling root-tip segments containing meristems (M), roots without root-tip meristems (R–M) and (because we did not observe GUS activity with most of the analysed promoters at 7 dpi) *G. rostochiensis*-infected root segments containing syncytia at 14 dpi (S). Different levels of expression of all the analysed genes were detected in roots as well as in syncytia. An increased level of transcript in meristems in comparison with the rest of the root (R–M) was observed only for the *NIK* gene (Fig. 3). In the case of the *CYP97A29*, *DFR* and *FLS* genes, the expression in root meristems was lower than in R–M. Transcript levels of the analysed genes varied in syncytia at 14 dpi. The level of the *PMEI* and *DFR* mRNAs was lower in syncytia than in other root parts, while expression of the *CYP97A29* and *NIK* genes was moderate and comparable with that observed in the M and R–M samples, respectively. The highest transcript abundance in syncytia was observed for the *FLS* gene and this was similar to its level in R–M samples.

## Discussion

The molecular mechanisms controlling changes occurring during the development of a NFS from a single initial cell into a multicellular syncytium are largely uncharacterized. There is a particular dearth of knowledge concerning the promoter *cis* regulatory elements responsible for the control of host gene expression during plant–nematode interactions. In this study we have examined the pattern of transcriptional regulation of five tomato genes selected from a panel of genes showing altered expression patterns during nematode parasitism (Swiecicka et al. 2009).

The activity of the promoter regions of the five selected genes (*CYP97A29*, *DFR*, *FLS*, *NIK* and *PMEI*) was analysed in tomato and potato hairy roots before and after infection with juveniles of *G. rostochiensis*. GUS activity was detected in different parts of the hairy roots depending on the gene promoter, but in all cases it was observed in or close to NFS. This finding and the results of the RT-PCR analysis clearly indicate that the host genes exploited by the nematode in NFS development are involved in common basic processes occurring in root tissues or in other plant organs.

The promoter most often used for the production of transgenic plants is that of the cauliflower mosaic virus (*CaMV*) 35S gene. In transgenic tobacco roots, the full 35S promoter was activated in 90 % of NFS induced by *M. incognita* and in 27 % of NFS induced by juveniles of *Globodera tabacum* subsp. *tabacum* (Bertioli et al. 1999). Urwin et al. (1997) showed that the 35S promoter was activated in the gall tissue surrounding the feeding site of *M. incognita.* Similarly, Goverse et al. (1998) found that expression of a GFP reporter gene fused to the 35S promoter was strongly upregulated in young feeding cells during infection by *G*. *rostochiensis*. However, these findings are not corroborated by results obtained in *Arabidopsis*, where 35S-driven GUS activity was down-regulated in NFS induced by *H. schachtii* (Goddijn et al. 1993; Sijmons et al. 1994). Moreover, Goddijn et al. (1993) also showed that the 35S promoter and other constitutive promoters of genes such as bacterial nopaline synthase, rooting loci (*rol*) and *T*-*cyt*, plant phenylalanine ammonia-lyase I and others were down-regulated in syncytia. Besides this discrepancy, constitutive promoters are not a good choice to drive the expression of resistance or other genes encoding nematode toxic compounds, because rather high root or syncytium specificity is essential.

Promoter tagging is one method that has been employed for the identification of NFS-specific promoters (Barthels et al. 1997; Favery et al. 2004). Barthels et al. (1997) analysed six tags that were differentially activated during the development of the NFS, and three of these were reintroduced as promoter-*gusA* fusions and analysed in detail. Besides the NFS, GUS activity was also detected in the roots, shoots and leaf vascular tissue of transformed *Arabidopsis* plants. Another approach used to identify NFS-specific promoters is the detailed analysis of regulatory sequences of genes identified as being differentially expressed in these structures.

The expression patterns of genes and the activities of most gene promoters analysed to date, have not been restricted to NFS or roots, e.g. *RPE* (Favery et al. 1998), *LEMMI9* (Escobar et al. 1999), *AtFH6* (Favery et al. 2004), or *AtAMT1;2*, *LBD41*, *ADF3* and *LTP* (Fuller et al. 2007), and *NtCel7* (Wang et al. 2007). The *RPE* gene, encoding d-ribulose-5-phosphate 3-epimerase, was found to be essential for the early steps of NFS formation induced by *M. incognita* and later induced by both root-knot and cyst nematodes, and to a lower level in syncytia. During root development, *RPE* is normally expressed in the meristems and lateral root primordia (Favery et al. 1998). The promoters of the *ADF3* and *LTP* genes, which are activated in different parts of uninfected *Arabidopsis* roots, showed activity in the galls of *M. incognita*, and during the intial steps of *H. schachtii* parasitism, but not when the female became saccate (Fuller et al. 2007). Besides NFS induced by *M. incognita*, the promoter of the *LEMMI9* gene, coding for a Lea-like protein, is strongly induced in roots and green tomato fruits (Van der Eycken et al. 1996). Escobar et al. (1999) located a 12-bp repeat that is possibly involved in the formation of DNA–protein complexes in the *LEMMI9* promoter, which might be related to transcriptional activation of the *LEMMI9* gene in the giant cells. Formin, encoded by the *AtFH6* gene, is required for organization of the actin cytoskeleton, and its promoter was found to be up-regulated in developing giant cells (Favery et al. 2004). GUS activity related to this promoter was observed in differentiating vascular cylinder cells just above the root-tip meristem, in the vascular tissue of the lateral root primordia and in the newly emerged lateral roots. In young *Arabidopsis* seedlings, low levels of *AtFH6* promoter activity were also detected in the vascular bundles of leaves and in the stipules (Favery et al. 2004).

Promoter deletion analysis has been used to define regulatory fragments that show specific activation. *TobRB7*, encoding a putative water channel (Conkling et al. 1990) that is expressed in root meristematic and immature vascular cylinder cells, was up-regulated in tobacco giant cells induced by *M. incognita* (Yamamoto et al. 1991; Opperman et al. 1994). Deletion of the *TobRB7* promoter sequence resulted in restriction of its activity only to NFS of *Meloidogyne*, but not of *G. tabacum* in tobacco (Yamamoto et al. 1991). The promoter of the *pyk20* gene from *A. thaliana* has also been analysed in detail (Puzio et al. 2000), and a regulatory region located between −277 and −1 bp relative to the start codon, encompassing the 5′ UTR, was found to be necessary to enhance the level of GUS expression in NFS. GUS activity was produced by all analysed *pyk20* promoter deletion clones in other organs of *Arabidiopsis* seedlings (Puzio et al. 2000). Analysis of the promoter of the *HS1* ^*pro*−1^ resistance gene from sugar beet identified *cis* elements responsible for NFS-specific gene expression located within the sequence between −255 and +247 bp relative to the transcriptional initiation site, whereas an enhancer region, active in sugar beet and *A. thaliana*, was located between −1,199 and −705 bp (Thurau et al. 2003).

The identification of a specific set of *cis* regulatory elements activated by different nematode species is a goal that has yet to be achieved. In the present study, the investigated gene promoters exhibited similar patterns of activity in different root tissues and were strongly up-regulated in syncytia induced by *G. rostochiensis* in tomato and potato roots. The hairy root system employed in this study is a simple and fast tool to test whether promoters are potentially useful for biotechnological applications. However, it is necessary to corroborate any findings by performing further analyses using plants with stably introduced transgenes.

The sequences of the promoters isolated for this study were analysed using bioinformatics tools and some known pathogenesis-related *cis* regulatory motifs were found. However, the function of these potential regulatory elements has so far only been confirmed for bacterial and fungal pathogens. Among the 29 classes of transcriptional regulators identified in *A. thaliana*, members of only three appear to function in the pathogen response: AP2/ERF (APETALA2/ETHYLENE-RESPONSIVE ELEMENT), WRKY and MYB (Riechmann 2002). The AP2/ERF and WRKY families are plant-specific. Some of them, like the W1 and W2-*box* [WRKY, (T)TGAC(C/T)], GCC-*like* (AP2/ERF, AGCCGCC), JERE (AP2/ERF, AGACCGCC) and S-*box* (AP2/ERF, AGCCACC) factors have been well described (Gurr and Rushton 2005). In *Arabidopsis*, there are 72 expressed *WRKY* genes that encode crucial regulators of the defence transcriptome and plant pathogen resistance (Eulgem and Somssich 2007). In many plant-pathogen models it has been demonstrated that WRKY transcription factors may function as positive or negative regulators of the plant defence network (Eulgem and Somssich 2007; Pandey and Somssich 2009). Recently, Grunewald et al. (2008) showed that *AtWRKY23* is involved in the development of syncytia induced by *H. schachtii*. *WRKY23* was shown to be strongly up-regulated in young syncytia, while its expression decreased during their further development. Activation of the *WRKY23* promoter is related to auxin accumulation and *WRKY23* acts downstream of the primary auxin response. Two paralogous genes of tomato, *SlWRKY72a* and *b*, were found to be up-regulated during the resistance response against root-knot nematode and potato aphids mediated by the *Mi*-*1* gene (Bhattarai et al. 2010). Similarly, the *Arabidopsis* orthologue *AtWRKY72* was also required for full basal defence against this nematode (Bhattarai et al. 2010). These results demonstrate that WRKY transcription factors are not only induced by bacterial or fungal pathogens, but also by nematodes. In the present study, we have identified putative *W*-*box* regulatory elements, that specifically bind WRKY proteins, in the promoters of each of the 5 genes whose expression is up-regulated by nematodes. The use of promoter deletion analysis and complementary methods is required to examine the role of the *W*-*box* and other putative elements in regulating the expression of these genes.

*Solanum tuberosum* is a close relative of *Solanum lycopersicoides* (Bohs and Olmstead 1997) and both are good hosts for *G. rostochiensis*. The similar activity patterns of the analysed promoters in syncytia induced in tomato and potato roots observed in the present study suggest that the manner of their regulation is the same in both species, and that it should be possible to use these promoters to control expression of anti-nematode products in related plants.

In conclusion, we have demonstrated that the promoters of 5 tomato genes (*CYP97A29*, *DFR*, *FLS*, *NIK* and *PMEI*) are active in syncytia induced by *G. rostochiensis* infection in the roots of both tomato and potato. These promoters may be used to drive the expression of nematocidal products in transgenic plants, but detailed functional characterization of their regulatory sequences, including deletion analysis, is required.

## Electronic supplementary material

Below is the link to the electronic supplementary material.

Supplementary material 1 (PDF 326 kb)

## Abbreviations

ITE: Independent transformation event
NFS: Nematode feeding site
