# Supplementary material for: Analysis of tomato gene promoters activated in syncytia induced in tomato and potato hairy roots by Globodera rostochiensis
Source: Transgenic Res. 2012 Nov 6;22(3):557–69. doi: 10.1007/s11248-012-9665-4 (PMC3653032; doi:10.1007/s11248-012-9665-4)

Analysis of tomato gene promoters activated in syncytia induced in tomato and potato hairy roots by *Globodera rostochiensis*

Wiśniewska A[a*], Dąbrowska-Bronk J[b], Szafrański K[a,d], Fudali S[c], Święcicka M[b], Czarny M[b], Wilkowska A[a], Morgiewicz K[a], Matusiak J[a], Sobczak M[c], Filipecki M[b]

[a]Department of Plant Physiology, Faculty of Agriculture and Biology, Warsaw University of Life Sciences (SGGW), Nowoursynowska 159, 02-776 Warsaw, Poland

[b]Department of Plant Genetics, Breeding and Biotechnology, Faculty of Horticulture and Landscape Architecture, Warsaw University of Life Sciences (SGGW), Nowoursynowska 159, 02-776 Warsaw, Poland

[c]Department of Botany, Faculty of Agriculture and Biology, Warsaw University of Life Sciences (SGGW), Nowoursynowska 159, 02-776 Warsaw, Poland

[d]present address: Université Pierre et Marie Curie, UMR 7138 (UPMC CNRS IRD MNHN), Systématique, Adaptation, Evolution (SAE), bâtiment A, 7, quai St Bernard, 75005 Paris, France

[*]corresponding author: anita_wisniewska@sggw.pl; Tel.: +48 22 5932533; fax: +48 22 5932521

Supplementary materials:

Table S1 Genes, homologues and primers used for the amplification of promoter fragments

Table S2. Primers used for subcloning promoter fragments into binary vector pCAMBIA1381Z

Table S3 Numbers of hairy roots derived from ITEs and used for GUS activity assays after infection by *G. rostochiensis* in each time point.

Table S4 Primer pairs used in RT-PCR assays

Table S5 Pathogen responsive *cis* regulatory elements identified in the analysed promoters with the PLACE algorithm.

Table S6 GUS activity in syncytia induced by *G. rostochiensis*

Fig. S1 Morphology and development of hairy roots in tomato cotyledon explants (**a**), potato leaf and stem explants (**b**), and tomato hairy roots 3 weeks after transformation with *A. rhizogenes* containing vector pCAMBIA1381Z(k) (**c**). Tomato explants transformed by wild-type *A. rhizogenes* on medium with (**d**) and without selection agent (**e**). Potato hairy roots 5 weeks after transformation of stem (**f**) and leaf explants (**g**). Tomato hairy roots 1 week after passage (**h**). Tomato hairy roots with lateral roots 2-3 weeks after passage (**i**). Scale bars – 10 mm.

Fig. S2 Morphological changes to control root after *G. rostochiensis* infection. Necrosis (arrows) caused by juvenile migration (1–7 dpi) **(a)**. Syncytium (star) (14 dpi) **(b)**. Syncytium (star) with adult female (arrow head) and males (black arrows) (40 dpi) **(c)**. Scale bars – 0.5 mm.

Table S1 Genes, homologues and primers used for the amplification of promoter fragments

Gene	DFCI Tomato Gene Index* (length of cDNA, bp)	NCBI Acc. no. of tomato nucleotide sequences **	Closest homologues and similarity level (blast x)	Gene-specific primers used for amplification of promoter regions (5’-3’)***
CYP97A29	TC236156 (2255)	EU849605	Cytochrome CYP97A29-type monooxygenase 97A29 [*Solanum lycopersicum*], ACJ25969, Expect = 0.0, Positives = 595/595 (100%)	CYP.GSP1: GTTCAACAGTAAATTCGCCTG<br>CYP.GSP2: TAATTCAGCTCGCCTTTTCTC
DFR	TC229037 (1275)	-	1. Rossmann-fold NAD(P)-binding domain-containing protein [*Arabidopsis thaliana*], NP_194455, Expect = 1e-107, Positives = 245/336 (73%),<br>2. Dihydroflavonol-4-reductase [*Zea mays*], NP_001150871, Expect = 2e-100, Positives = 238/334 (71%)	DFR.GSP1: GACTTCTCAGGATGGCGCAC<br>DFR.GSP2: TCTCCTCCATGATACAAAATT
FLS	TC221782 (1193)	AK323212.1<br>AK246757.1	Putative flavonol synthase [*Arabidopsis thaliana*], AAM14878, Expect = 8e-59, Positives = 190/352 (54%)	FLS.GSP1: CGACGGTGGGGATTGAGTTT<br>FLS.GSP2: GTGGCTCAGATTCAAGGGGA
NIK	TC218759 (1783)	-	Protein kinase domain-containing protein [*Arabidopsis thaliana*], NP_186798, Expect = 5e-178, Positives = 330/406 (81%)	NIK.GSP1: CTCCTTTGTTACTCTTTTGTTT<br>NIK.GSP2: TGCTCTCACAAAACCCACTTC
PMEI	TC221715 (834)	AK246520.1<br>AK320939.1	Plant invertase/pectin methylesterase inhibitor domain-containing protein [*Arabidopsis thaliana*], NP_197574, Expect = 8e-43, Positives = 122/162 (75%)	PMEI.GSP1: TGGGTGTGTCCGAGTATGAA<br>PMEI.GSP2: GTGAATGTATGTGTTGGTAG

* http://compbio.dfci.harvard.edu/tgi/ (07.10.2011); ** if available; *** AP1: GTAATACGACTCACTATAGGGC, AP2: ACTATAGGGCACGCGTGGT – forward primers.

Table S2. Primers used for subcloning promoter fragments into binary vector pCAMBIA1381Z

Gene	Primers* (5’-3’)	Restriction sites added to primers
CYP97A29	5’GAGAATTCACTATAGGGCACGCGTGGT3’<br>5’CAGGATCCGAATGAGAAAAAGATGGAGATAATG3’	*Eco*RI<br>*Bam*HI
DFR	5’GAGAATTCACTATAGGGCACGCGTGGT3’<br>5’TAGAGGATCCGATACAAAATTTCTTCTTGC3’	*Eco*RI<br>*Bam*HI
FLS	5’CAGGATCCACTATAGGGCACGCGTGGT3’<br>5’CAAAGCTTGTGGCTCAGATTCAAGGGATAA3’	*Bam*HI<br>*Hind*III
NIK	5’GAGAATTCACTATAGGGCACGCGTGGT3’<br>5’CAGGATCCACTCGATTAATTGATTTTGAAACAC3’	*Eco*RI<br>*Bam*HI
PMEI	5’GAGAATTCACTATAGGGCACGCGTGGT3’<br>5’TCGAGGATCCGTGAATGTATGTGTTGGTAG3’	*Eco*RI<br>*Bam*HI

* restriction sites added to facilitate subcloning are underlined.

Table S3 Numbers of hairy roots derived from ITEs and used for GUS activity assays after infection by *G. rostochiensis* in each time point.

Gene promoter	Number of analysed ITEs	
	Tomato	Potato
CYP97A29	4	6
DFR	12	7
FLS	5	9
NIK	4	5
PMEI	7	9

Table S4 Primer pairs used in RT-PCR assays

Gene	Forward primer	Reverse primer	Annealing temperature (°C)
CYP97A29	CCAGTTTGGGAGTTGCCTAT	GCTTGGCCATGACACTAGGT	64
DFR	AACAGGAGGAACAGGCTTCA	TGAAATAGGCCAATGCAACC	56
FLS	CTAATATTTATCCCCTTGAAT	GCACAAAGACCTTAAGATAG	56
NIK	TGTATTGACAGCCATGGCGG	CCAAGCATGTTTAATGGGGA	62
PMEI	CGAGCAGGTAAAGCGTCTG	AGCAACATTCGTAATCTTTCG	56
UBI3 (X58253.1)	ACCACCACGGAGACGGAG	ATGCAGATCTTCTGAAAAC	58

Table S5 Pathogen responsive *cis* regulatory elements identified in the analysed promoters with the PLACE algorithm.

***Cis* regulatory element**	**Gene**	**Positions ***	***Cis* regulatory element**	**Gene**	**Positions ***
**BIHD1*Os* (TGTCA)**	*PMEI*	-710(+), -933(+)	**SEBFcons *St* PR10A (YTGTCWC)**	*PMEI*	-61(+)
	DFR	-344(-), -16(-), -963(-), -361(+), -399(+), -492(+)		*DFR*	-415(-)
	CYP97A29	-231(-), -583(-), -1222(+), -319 (-), -1581(-)		*CYP97A29*	-
	FLS	-228(-), -910(+)		*FLS*	-
	NIK	-126(-), -140(-), -762(-), -773(-), -802(-), -995(-)		*NIK*	-139(-), -801(-)
**CACGTG *motif* (CACGTG) (G-box)**	*PMEI*	-	**WB *box* *Pc*WRKY1 (TTTGACY)**	*PMEI*	-671(-)
	DFR	-407(-), -407(+)		*DFR*	-
	CYP97A29	-323(-), -323(+)		*CYP97A29*	-
	FLS	-		*FLS*	-1270(-), -653(+)
	NIK	-		*NIK*	-714(+), -826(+)
**ELRE*corePc*RP1 (TTGACC)**	*PMEI*	-672(-)	**W *box At*NPR1 (TTGAC)**	*PMEI*	-672(-), -709(-), -1070(+)
	DFR	-		*DFR*	-360(-), -964(+)
	CYP97A29	-		*CYP97A29*	-318(-), -584(+)
	FLS	-1271(-)		*FLS*	-1271(-), -410(+), -654(+)
	NIK	-110(-), -714(-), -826(-)		*NIK*	-111(+), -127(+), -715(+), -827(+), -905(+)
**GT-1*Gm*SCaM4 (GAAAAA)**	*PMEI*	-265(-), -361(+), -1213(-)	**W *box Hv*ISO1 (TGACT)**	*PMEI*	-
	DFR	-		*DFR*	-142(-), -176(-), -744(-)
	CYP97A29	-7(-), -391(-), -1021 (-), -1126(-), -1316(-), -500(+), -1048(+), -1168(+), -1347(-)		*CYP97A29*	-335(+), -526(+)
	FLS	-329(-), -675(-), -1031(-), -1125(-), -1139(-), -1504(-), -528(+), -779(+)		*FLS*	-53(-), -446(-), -653(+)
	NIK	-53(-), -84(-), -265(-), -295(-), -616(-), -194(+), -220(+), -446(+)		*NIK*	-
**OSE1root nodule (AAAGAT)**	*PMEI*	-760(+), -1220(-)	**W *box Nt*ERF3 (TGACY)**	*PMEI*	-673(-)
	DFR	-551(+), 902(+)		*DFR*	-142(-), -176(-), -744(-), -819(-), -502(+)
	CYP97A29	-10(-), -101(-), -1042(-)		*CYP97A29*	-335(+), -526(+), -1601(+)
	FLS	-		*FLS*	-53(-), -446(-), -519(-), -1272(-), -653(+),
	NIK	-		*NIK*	-505(-), -110(+), -486(+), -714(+), -826(+), -857(+)
**OSE2 root nodule (CTCTT)**	*PMEI*	-72(+), -243(-), -543(-), -1111(+)	**WRKY71*Os* (TGAC)**	*PMEI*	-349(-), -673(-), -710(-), -933(-), -1070(-)
	DFR	-286(-), -836(-), -66(+), -147(+)		*DFR*	-142(-), -176(-), -361(-), -399(-), -492(-), -744(-),

			-819(-), -345(+), -417(+), -503(+), -964(+)
CYP97A29	-1251(-), -26(+), -398(+), -1657(+)	*CYP97A29*	-319(-), -1581(-), -232(+), -336(+), -512(+), -527(+), -584(+), -1223(+), -1602(+)
FLS	-63(+), -1328(+)	*FLS*	-53(-), -446(-), -519(-), -910(-), -1272(-), -229(+), -410(+), -654(+)
NIK	-30(-), -93(-), -307(+)	*NIK*	-505(-), -111(+), -127(+), -141(+), -487(+), -544(+), -702(+), -715(+), -763(+), -774(+), -803(+), -827(+), -858(+), -905(+), -956(+)

(+) – sense strand, (-) – antisense strand; W=A/T, Y=T/C, * positions relative to ATG start codon

References:

Boyle B, Brisson N. Repression of the defense gene PR-10a by the single-stranded DNA binding protein SEBF. Plant Cell 13: 2525-2537 (2001)

Buchel AS, Brederode FT, Bol JF, Linthorst HJM. Mutation of GT-1 binding sites in the Pr-1A promoter influences the level of inducible gene expression in vivo. Plant Mol Biol 40:387-396 (1999)

Chakravarthy S, Tuori RP, DAscenzo MD, Fobert PR, Despres C, Martin GB. The tomato transcription factor Pti4 regulates defence-related gene expression via GCC box and non-GCC box cis elements. Plant Cell 15: 3033-3050 (2003)

Eulgem T, Rushton PJ, Schmelzer E, Hahlbrock K, Somssich IE. Early nuclear events in plant defence signalling: rapid gene activation by WRKY transcription factors. EMBO J. 18:4689-4699 (1999)

Fehlberg V, Vieweg MF, Dohmann EM, Hohnjec N, Puhler A, Perlick AM, Kuster H. The promoter of the leghaemoglobin gene VfLb29: functional analysis and identification of modules necessary for its activation in the infected cells of root nodules and in the arbuscule-containing cells of mycorrhizal roots. J Exp Bot. 56:799-806 (2005)

Laloi C, Mestres-Ortega D, Marco Y, Meyer Y, Reichheld JP. The Arabidopsis cytosolic thioredoxin h5 gene induction by oxidative stress and its W-box-mediated response to pathogen elicitor. Plant Physiol. 134:1006-1016 (2004)

Luo H, Song F, Goodman RM, Zheng Z. Up-regulation of OsBIHD1, a rice gene encoding BELL homeodomain transcriptional factor, in disease resistance responses. Plant Biol (Stuttg). 7: 459-468 (2005)

Nishiuchi T, Shinshi H, Suzuki K. Rapid and transient activation of transcription of the ERF3 gene by wounding in tobacco leaves: Possible involvement of NtWRKYs and autorepression. J Biol Chem. 279: 55355-55361 (2004)

Rushton PJ, Torres JT, Parniske M, Wernert P, Hahlbrock K, Somssich IE. Interaction of elicitor-induced DNA-binding proteins with elicitor response elements in the promoters of parsley PR1 genes. EMBO J. 15:5690-5700 (1996)

Sun C, Palmqvist S, Olsson H, Boren M, Ahlandsberg S, Jansson C. A novel WRKY transcription factor, SUSIBA2, participates in sugar signaling in barley by binding to the sugar-responsive elements of the iso1 promoter. Plant Cell 15: 2076-2092 (2003)

Xu X, Chen C, Fan B, Chen Z. Physical and Functional Interactions between Pathogen-Induced Arabidopsis WRKY18, WRKY40, and WRKY60 Transcription Factors. Plant Cell 18:1310-1326 (2006)

Table S6 GUS activity in syncytia induced by *G. rostochiensis*

Promoter	ITE	No. of Petri dishes with hairy roots	No. of GUS positive syncytia	Total no. of syncytia	Percentage of GUS-positive syncytia
CYP97A29	5	12	41	64	64
FLS	4	13	43	73	59
PMEI	7	15	82	139	59

Fig. S1 Morphology and development of hairy roots in tomato cotyledon explants (**a**), potato leaf and stem explants (**b**), and tomato hairy roots 3 weeks after transformation with *A. rhizogenes* containing vector pCAMBIA1381Z(k) (**c**). Tomato explants transformed by wild-type *A. rhizogenes* on medium with (**d**) and without selection agent (**e**). Potato hairy roots 5 weeks after transformation of stem (**f**) and leaf explants (**g**). Tomato hairy roots 1 week after passage (**h**). Tomato hairy roots with lateral roots 2-3 weeks after passage (**i**). Scale bars – 10 mm.

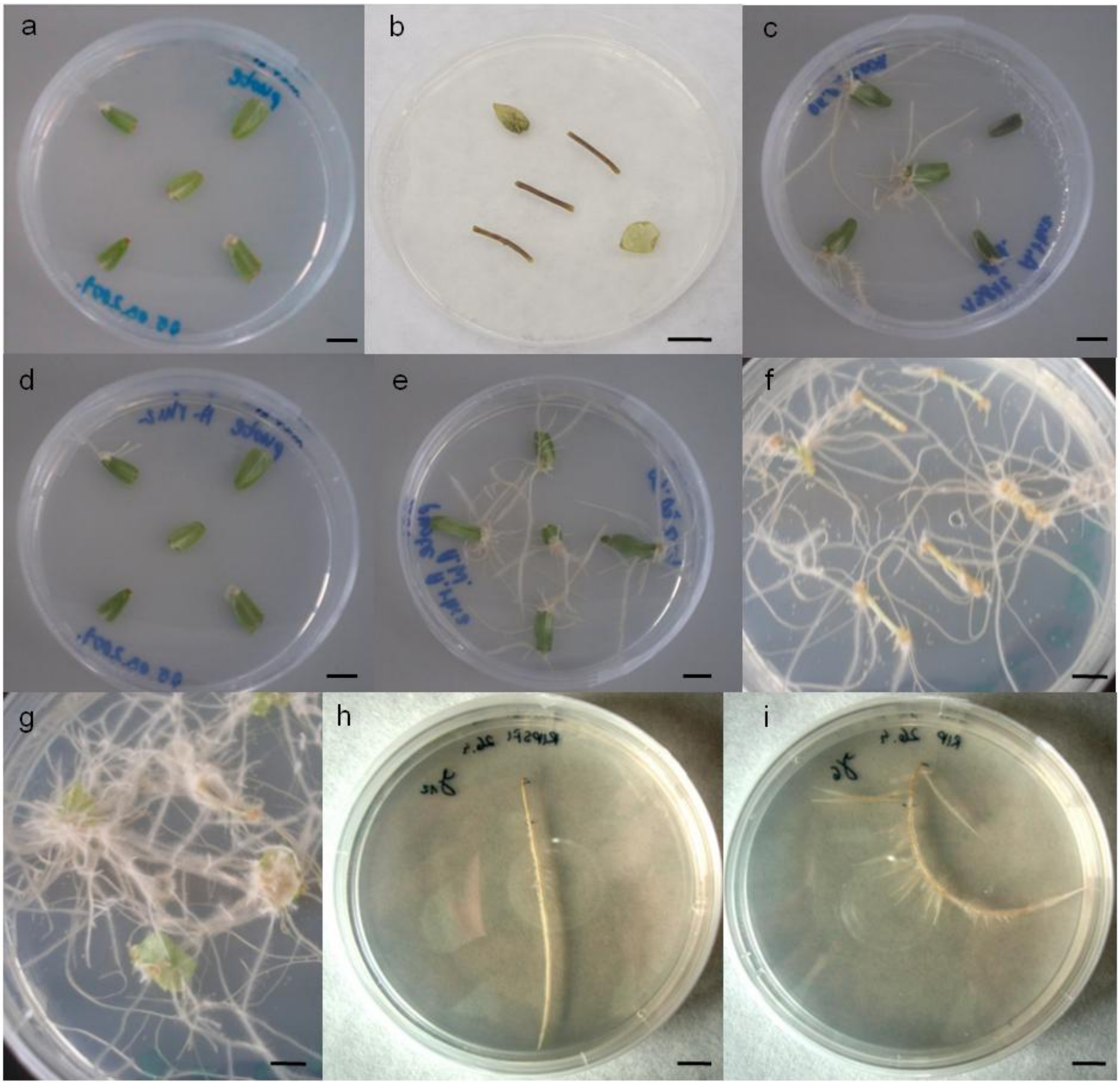

Fig. S2 Morphological changes to control root after *G. rostochiensis* infection. Necrosis (arrows) caused by juvenile migration (1–7 dpi) **(a)**. Syncytium (star) (14 dpi) **(b)**. Syncytium (star) with adult female (arrow head) and males (black arrows) (40 dpi) **(c)**. Scale bars – 0.5 mm.

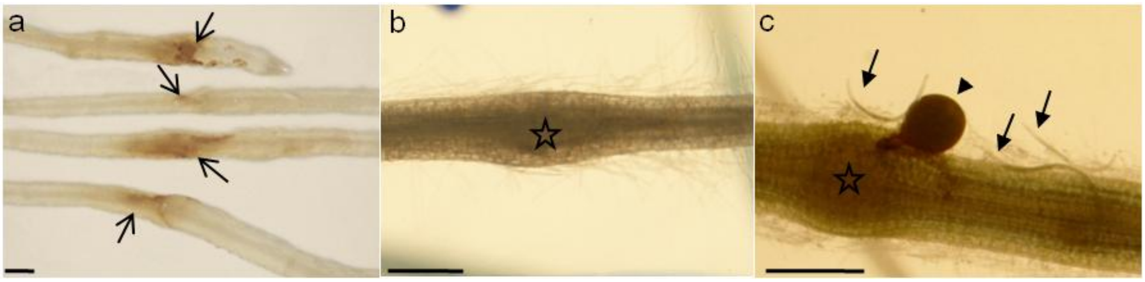

Supplement: Supplementary file 1 — Supplementary material 1 (PDF 326 kb) [file 11248_2012_9665_MOESM1_ESM.pdf]
